# Validation of the portable MX3 hydration testing system for measuring saliva osmolality

**DOI:** 10.3389/fnut.2026.1882490

**Published:** 2026-08-25

**Authors:** A. Maleah Winkler, Mitchell E. Zaplatosch, Caroline J. Smith, William M. Adams, Charlotte F. Willis, Skylar Bovine, M. Ryan McClure, Genesis Santos, Yana Chmunevich, Samantha J. Mitchell, Emilee O'Grady, William Kinnaird

**Affiliations:** 1 Department of Kinesiology, Augusta University, Augusta, GA, United States; 2 Department of Exercise Science and Sports Management, Kennesaw State University, GA, United States; 3 Department of Kinesiology, Appalachian State University, Boone, NC, United States; 4 Department of Kinesiology, Michigan State University, East Lansing, MI, United States; 5 Department of Kinesiology, University of North Carolina at Greensboro, Greensboro, NC, United States; 6 School of Sport, Exercise and Health Sciences, National Center for Sport and Exercise Medicine (NCSEM), Loughborough University, Loughborough, United Kingdom

**Keywords:** dehydration, field assessment, osmolarity, osmometer, reliability, validity

## Abstract

Saliva, specifically salivary osmolality, has gained increasing interest as a non-invasive hydration marker providing rapid results that can be easily obtained in a field setting. However, it is unclear as to whether commercially available hydration devices can accurately measure salivary osmolality. Thus, this study assessed the validity of the MX3 Portable Hydration device against a laboratory-grade freezing-point depression benchtop osmometer (i.e., criterion measure). A total of eight saliva samples were collected from 10 male participants (total saliva samples, *n* = 80; age, 25 ± 5 y; mass, 79.2 ± 12.1 kg; height, 181.1 ± 6.6 cm) across a two-day period. Samples were stored at 4°C before being centrifuged at 3,000 rpm for 1 min. Samples were simultaneously analyzed via freezing-point depression osmometry (250 μL per sample) and MX3 portable device (~10 μL per sample). Samples were analyzed in duplicate or triplicate, depending on available volume. Results demonstrated moderate agreement between methods, based on Lin's concordance correlation coefficient (CCC = 0.91), and the MX3 demonstrated excellent reliability across repeated measurements of the same sample [ICC (1, 2) = 0.991, (0.988, 0.992)]. A significant proportional bias indicated the MX3 overestimated salivary osmolality values by 24% at the low end [bias = 1.24, 95% CI (1.15, 1.33)] but underestimated osmolality values at higher osmolality values by 25% [bias = 0.75, 95% CI (0.62, 0.90)] when compared to the criterion measure. However, the MX3 demonstrated good validity at mid-range values (average 80 mOsm/kg), where bias decreased to 9% [bias = 1.09, 95% CI (1.05, 1.14)], with LOA from 0.77 to 1.54. These data suggest the MX3 portable device is a reasonable field measure of saliva osmolality, providing a reliable and non-invasive hydration assessment marker for field applications.

## Introduction

Current hydration assessment methods are multi-factorial and include quantification of biologic specimens (e.g., urinary and hematologic hydration measures), changes in body mass, and perceptions of thirst. Each of these current methods, while used to assess hydration status, has contextual, access, equipment, and/or temporal limitations, thus rendering a single assessment method or biologic sample not useful across all settings and scenarios (1). There is increasing interest in the use of saliva as a non-invasive and accessible body fluid for rapid hydration assessment, especially in field-based settings (2).

Salivary osmolality increases as total body water decreases and extracellular fluids become more concentrated (3). As exercise intensifies, sympathetic dominance and reduced parasympathetic input lead to a more concentrated saliva, thereby increasing osmolality (3). This physiological response enhances the sensitivity of saliva as a tool for detecting dehydration during physical activity, particularly as levels of dehydration approach 2% body mass loss (4). Saliva osmolality has been shown to be as sensitive as both urine osmolality (5) and serum osmolality (6) for detecting hydration changes. However, high intra- and inter-individual variability raises questions regarding the sensitivity of saliva osmolality and low reliability and poor-to-weak predictive value as a hydration marker (from 3–6% dehydration, sensitivity = 64%, dropping to 42% with >6% dehydration) (7, 8).

There are limited options in terms of portable hydration assessment devices, which typically focus on wearable sweat monitoring and estimations of hydration status (e.g., the hDrop, Nix Hydration Biosensor, GX Sweat Patch, etc.). Other systems utilize salivary samples to assess hydration status via salivary osmolality, with varying results (5, 7–9). The MX3 Hydration Testing System, a portable device that provides real-time assessment of saliva osmolality, has been internally validated against a criterion measure (10). However, reliability results are mixed (7, 9, 11, 12). Winter et al. reported moderate-to-good reliability of the MX3 (ICC = 0.75) and an MDC90 of 21.3 mOsm, indicating that changes in MX3-measured salivary osmolality greater than 21.3 mOsm are likely to represent true changes rather than measurement error (9). The MX3 Hydration Testing System and laboratory freezing-point depression osmometers quantify salivary osmolality using different measurement principles. Freezing-point depression osmometry is widely considered the criterion laboratory method and determines osmolality by measuring the reduction in a sample's freezing point caused by dissolved solutes. The result reflects the total concentration of osmotically active particles in solution (6). In contrast, the MX3 uses an electrochemical sensing approach. This technique estimates salivary osmolality from the electrical properties of the saliva sample, which may be influenced by factors such as electrolyte composition, protein concentration, viscosity, and salivary flow rate (5, 13). These methodological differences may become important during dehydration, when changes in saliva composition accompany increases in salivary osmolality (3).

The MX3 is particularly advantageous for field-based hydration assessment due to its portability, rapid analysis, low sample volume requirements, and immediate availability of results (9, 10). On the other hand, freezing-point depression osmometry requires specialized laboratory equipment, greater sample volumes, and trained personnel (6, 10). The practical advantages of portable salivary assessment are appealing for athletic, occupational, military, and first-responder settings; however, differences in measurement technology may affect agreement across the range of salivary osmolality values (5, 13). Independent validation of the MX3 against a criterion laboratory osmometer is necessary to confidently use the device in applied settings.

Evidence on the M3′s performance remains limited. Existing studies have primarily focused on diagnostic classification of hydration status, associations between MX3-derived values and other hydration markers, or the device's reliability (7, 9, 11, 12). However, relatively little information is available regarding the criterion validity of the MX3 and the extent to which MX3-derived salivary osmolality values agree with those obtained using a laboratory-grade freezing-point depression osmometer. Therefore, independent validation of the MX3 against a criterion laboratory method is warranted. Thus, the purpose of this study was to formally validate the MX3 Portable Hydration system against a criterion standard freezing-point depression osmometer. We hypothesized that there would be good agreement between saliva osmolality measurements from the MX3 device and the benchtop freezing-point depression osmometer.

## Methods

### Experimental design

This prospective repeated-measures method-comparison design study evaluated the validity of the MX3 Portable Hydration system (MX3 LAB handheld device, MX3 diagnostics, Austin, TX, USA) against a criterion standard (The Advanced Osmometer Model 3250, Advanced Instruments, Norwood, MA, USA) across multiple sampling timepoints. Saliva samples were collected from each participant over a two-day period at four standardized time points for a total of 8 samples collected from each participant. Written informed consent was voluntarily obtained from all participants in advance of sample collection. The study was approved by Augusta University's Institutional Review Board (IRBNet ID# 2047457), and all procedures performed followed institutional guidelines.

### Participants

Ten male recruit firefighters (mean ± SD; age, 25 ± 5 y; body mass, 79.2 ± 12.1 kg; height, 181.1 ± 6.6 cm; BMI, 24.2 ± 3.7 kg·m^2^) from a local fire department in the southeastern part of the United States participated in this study. Inclusion criteria included being currently employed by the local Fire Department and being 18 years or older.

### Experimental protocol

**Figure 1:**
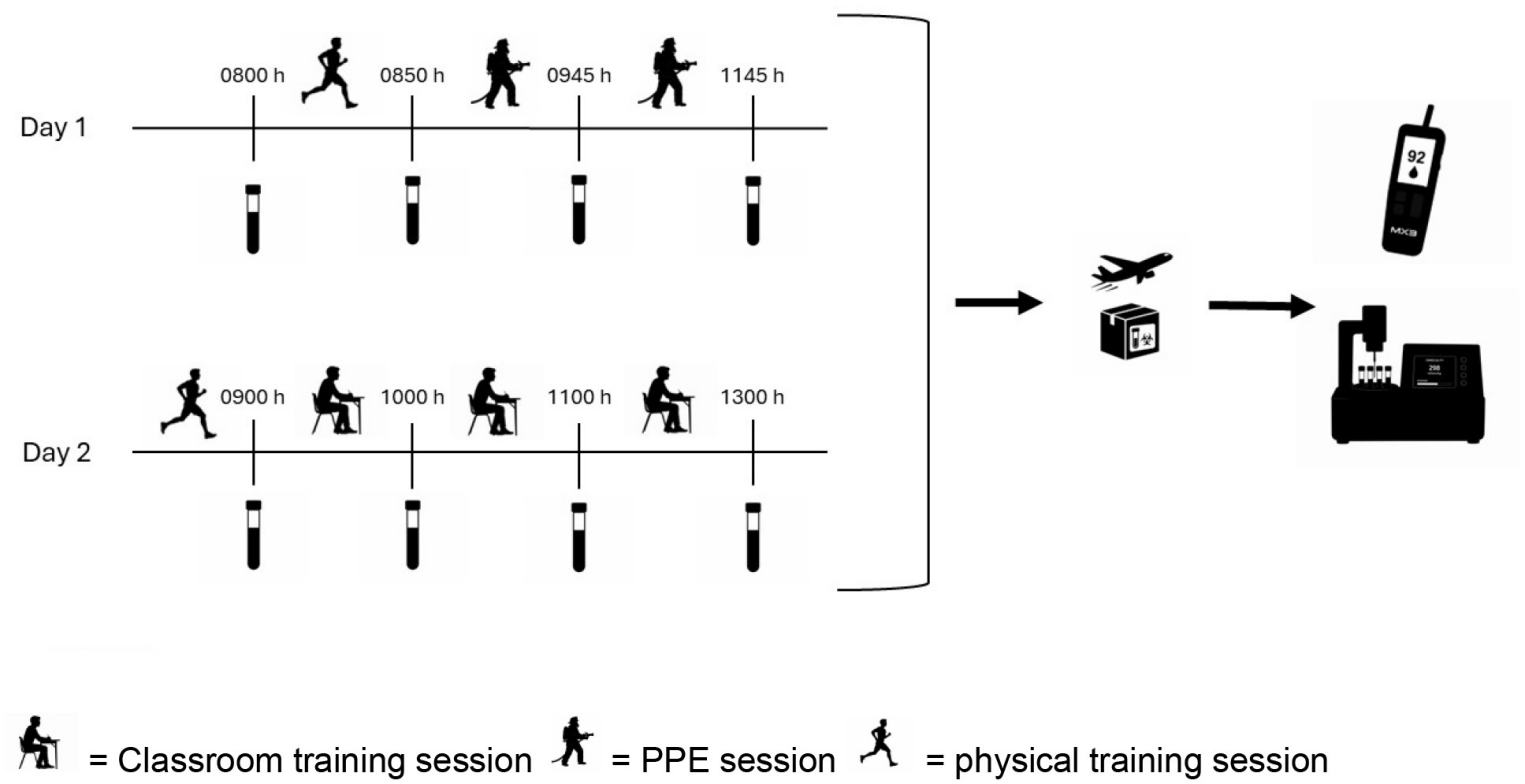
Saliva sampling timeline across two Fire Academy training days. Eighty samples were collected at multiple timepoints collected at different activity intensities. Samples were refrigerated during collection and shipped on ice for osmolality analysis comparison between the MX3 system and benchtop osmometer.

Eighty (80) saliva samples were collected over a period of two days during the last two weeks of Fire Academy (November-December 2025, Georgia). Samples were collected at different activity intensities, capturing within-day and between-day variability and allowing evaluation of device validity across hydration states (Figure 1). Participants were asked to refrain from smoking, eating or drinking for 10 min before providing the sample. Saliva samples were collected in 1.7 mL tubes (VWR Microcentrifuge Tubes, VWR International, Radnor, Pennsylvania, USA) filled with at least 0.25 mL of saliva. Participants actively spit into the tube as the method of collection.

Saliva collection timepoints during the first day of sampling included: (1) 8:00 AM, baseline prior to physical activity/ Physical Training (PT) session or wearing personal protective equipment (PPE), (2) 8:50 AM, directly after a morning PT session in PPE (excluding the helmet and air tank), (3) 9:45 AM, directly after completing difficult skills test until air tank depletion, and (4) 11:45 AM, immediately after completing multiple physical drills and then removing PPE.

The second day of sampling occurred one week later during classroom training. Four samples were taken at time points spaced 1 h apart. The first sample occurred at 9:00 AM, after PT. The following three samples occurred at 10:00 AM, 11:00 AM, and 12:00 PM. All four samples on day two were taken at rest, sitting in the classroom, without wearing PPE. On both data collection days, saliva samples were refrigerated during the data collection period and then shipped overnight on ice for osmolality measurements.

After removal from cold storage (4°C), samples were centrifuged at 3,000 rpm for 1 min. Samples were then simultaneously analyzed using both a benchtop freezing-point depression osmometer and the portable MX3 osmometer. Using a micropipette, 250 μL of saliva from each sample was transferred to a Safe-lock microcentrifuge tube for analysis with the benchtop osmometer. Samples were run in duplicate or triplicate, depending on the available sample volume. For saliva osmolality testing using the MX3 device, it was set to local mode, and 10 μL of each saliva sample was pipetted directly onto the test strip. Tests were run in triplicate if the sample volume was adequate. All sample outputs were recorded into a spreadsheet immediately after readout.

### Statistical analysis

Salivary osmolality was compared between devices using established methods of agreement. Each test of agreement was performed with the entire sample and with the exclusion of samples thought to interfere with the measurement according to the MX3 manufacturer guidelines [i.e., the overt presence of bubbles in the sample (*n* = 1)], as well as comparing samples that were sufficiently captured within only two measurements (duplicates) or three measurements (triplicates), vs. the whole dataset which included up to five measurements if sample volume permitted. Lin's concordance correlation coefficient (CCC) was calculated for the devices (14), which has been defined by agreement cutoffs ranging from poor (< 0.90), moderate (0.90–0.95), substantial (0.95–0.99), and almost perfect (>0.99) (15). Additional agreement measures comparing the benchtop osmometer to MX3 included constant error (CE = actual – predicted), root mean square error (RMSE = √Σ[predicted-actual]2/n), and standard error of the estimate (SEE = √Σ[predicted-actual] × √1 – r2), where in each equation “actual” was represented by the mean osmolality from the benchtop osmometer and “predicted” was represented by the mean osmolality measured using the MX3 device. Agreement between devices was also assessed and visualized using Bland Altman's 95% limits of agreement (LOA) technique (mean bias ± [1.96 x SD of differences]). The normality of residuals was assessed visually via normal quantile plots and with the Shapiro-Wilk test (16), while homogeneity of variance was assessed visually and with the Bresuch-Pagan test (17). All Bland-Altman analyses were evaluated for proportional bias by regressing the difference between devices on the mean of both devices. Values were log-transformed in the event of a normality violation, and more conservative limits of agreement were applied to the agreement range using the MOVER limits of agreement established by Donner and Zou (18). Where proportional bias was present, regression-based LOA were calculated at the lower, middle, and upper ends of the observed measurement range. Agreement between each device was further assessed using all trials via a linear mixed model to determine the ICC for absolute agreement [ICC (A,1)] by accounting for the greater number of trials conducted using the MX3 device for some measurements. ICC for consistency was calculated separately for the MX3 trials to determine the reliability of the device using a one-way random-effects ICC (ICC (1,k). Analyses were performed using R Studio with the “SimplyAgree” package (19), where statistical significance was set at *p* < 0.05.

## Results

**Figure 2:**
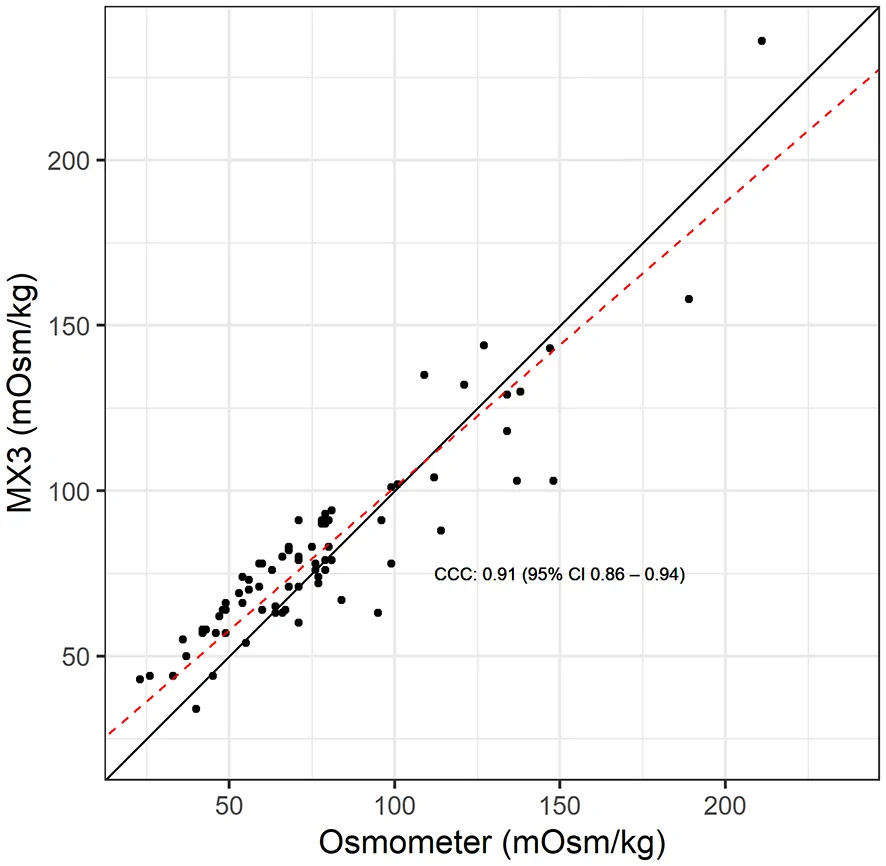
Lin's concordance correlation (CCC) between Osmometer and MX3 derived measures of osmolality. Dashed line represents the observed linear relationship between devices. The solid 45-degree line represents the theoretical perfect correlation between devices.

**Figure 3:**
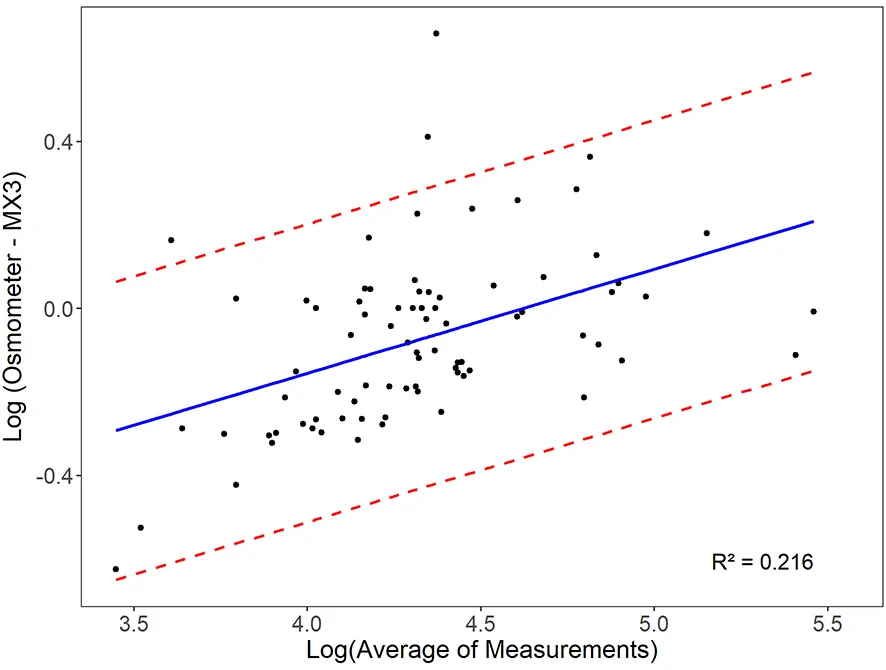
Bland-Altman plot displaying agreement between Osmometer and MX3. Values log-transformed to accommodate violation of the normality assumption. Upper and lower dashed red lines in the plot represent upper and lower 95% MOVER limits of agreement between measures. The middle blue line represents the relationship between the combined mean of each measurement pair and the difference between methods. Proportional bias was observed; therefore, the mean difference changes with increasing saliva concentration.

**Figure 4:**
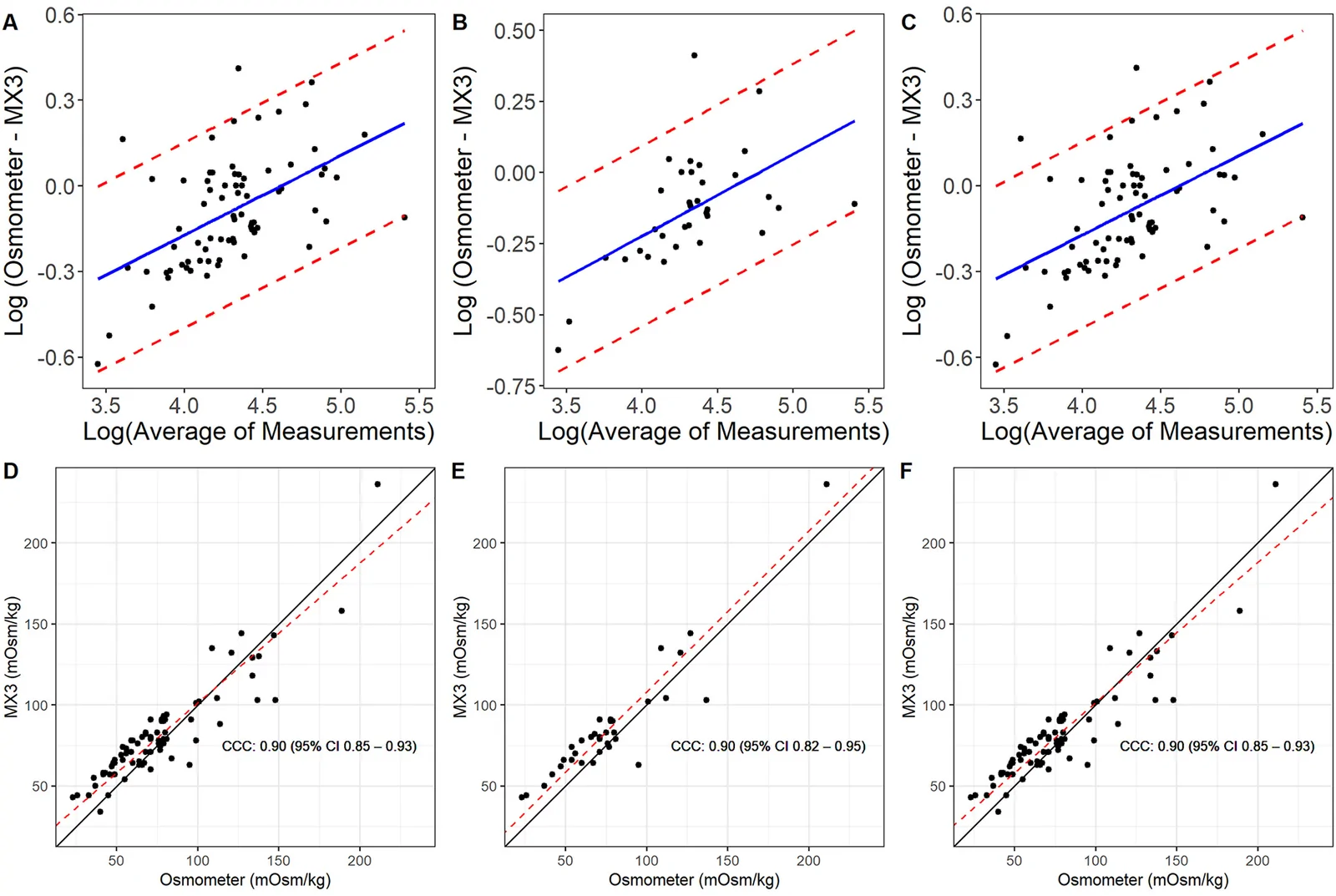
Bland-Altman Limits of Agreement **(A–C)** and Lin's Concordance Correlations **(D–F)** (CCC) for samples measured in Duplicate, Triplicate, or with samples containing bubbles removed (*n* = 1), respectively. In Bland-Altman plots, values were log-transformed to accommodate violation of the normality assumption. Upper and lower dashed red lines in the plot represent upper and lower 95% MOVER limits of agreement between measures. The middle blue line represents the relationship between the combined mean of each measurement pair and the difference between methods. Proportional bias was observed; therefore, the mean difference changes with increasing saliva concentration. In Lin's Concordance Correlation plots, the dashed line represents the observed linear relationship between devices. The solid 45-degree line represents the theoretical perfect correlation between devices.

For the full sample [80 available pairs, [Mean ± SD (Min, Max), mOsm/kg]: Osmometer, 80 ± 39, (23, 234); MX3, 83.5 ± 35 (34, 236)], agreement between methods was moderate based on CCC (Figure 2), while ICC was excellent [ICC (A, 1) = 0.91, 95% CI (0.86, 0.94)]. For the raw data, Bland-Altman analysis indicated a mean bias of −3.5 mOsm/kg [−6.9, −0.07], with lower LOA of −33.6 mOsm/kg and Upper LOA 26.6 mOms/kg. However, there was significant proportional bias (R2 = 0.216, *p* = 0.013) and a normality violation based on the Sharpiro-Wilk test (*p* < 0.001); thus, log-transformed bias and limits of agreement were adjusted for this bias and estimated at the lower, middle, and upper ends of the observed measurement range (Figure 3). At low average osmolality values (33 mOsm/kg), the MX3 overestimated the osmometer by 24% [bias = 1.24, 95% CI (1.15, 1.33)], with LOA ranging from 0.88 to 1.75. At mid-range values (average 80 mOsm/kg), bias decreased to 9% [bias = 1.09, 95% CI (1.05, 1.14)], with LOA from 0.77 to 1.54. At higher osmolality values (224 mOsm/kg), the MX3 underestimated the osmometer by approximately 25% [bias = 0.75, 95% CI (0.62, 0.90)] with narrower LOA from 0.53 to 1.06 (Figure 3). Additional statistics supported good agreement between measures (CE = −3.5 mOsm/kg, RMSE = 13.8 mOsm/kg, SEE: 13.9 mOsm/kg), but these values should be interpreted cautiously as they are based on an assumption of constant bias and uniform error variance across the measurement range. Sensitivity analyses revealed relatively consistent agreement (CCC = 0.9) regardless of which samples were included (duplicate samples only, triplicate samples only, samples with bubbles removed) (Figure 4). The MX3 device also demonstrated excellent within-sample reliability for a single trial [ICC (1, 1) = 0.982, 95% CI (0.978, 0.984)], which improved when averaging two trials [ICC (1, 2) = 0.991, 95% CI (0.988, 0.992)] and three Trials [ICC (1, 3) = 0.994, (0.992, 0.99)].

## Discussion

The purpose of this study was to determine the validity of measuring saliva osmolality with the MX3 portable hydration testing system compared to a laboratory-grade freezing-point depression osmometer as the criterion reference. Samples were collected from recruit firefighters over a two-day period in varied activity and environmental conditions. The primary finding of this study was that the MX3 device demonstrated moderate agreement with the benchtop osmometer (CCC = 0.91). A small constant mean error [−3.5 mOsm/kg, 95% CI (−6.9, −0.1)] was found with the MX3 compared to the benchtop osmometer, indicating that the MX3 measured slightly higher osmolality values on average. However, the practical significance of this average bias should be interpreted in the context of the proportional bias observed across the measurement range, with agreement worsening at both lower and higher salivary osmolality values. Additionally, the MX3 demonstrated excellent within-sample reliability (ICCs = 0.98–0.99), indicating that the MX3 provides consistent readings under the same conditions; however, further research is needed to determine if it can accurately detect changes in hydration status over time. The minimal detectable change reported by Winter et al. (9) indicates within-device measurement reliability and should not be interpreted as a threshold for acceptable agreement between the MX3 and a laboratory osmometer. In contrast, Zhao et al. (7) reported low day-to-day reliability (ICC = 0.20–0.39) for the MX3 system and poor diagnostic accuracy (sensitivity 0.09-0.57, specificity 0.63–0.82) when saliva osmolality was compared to urine specific gravity and body mass loss when measured at rest and following exercise in the heat. The discrepancies between our findings may be attributed to differences in study design as Zhao et al. (7) measured day-to-day reliability and diagnostic hydration classification between salivary osmolality and known measures of hydration status (e.g., body mass loss and urine specific gravity), whereas we only determined that the MX3 accurately measured salivary osmolality when compared to a criterion standard (i.e., benchtop osmometer) and that repeated measures of the same sample were reliable.

A significant proportional bias was found in this study (R2 = 0.213, *p* = 0.013) as agreement between devices decreased at the low and high osmolalities. The MX3 overestimated osmolality relative to the benchtop osmometer at lower values [+24% (bias = 1.24, 95% CI (1.15, 1.33)) at 33 mOsm/kg], demonstrated minimal bias at mid-range values [+9% (bias = 1.09, 95% CI (1.05, 1.14)) at ~80 mOsm/kg], and underestimated osmolality at higher values [−25% (bias = 0.75, 95% CI (0.62, 0.90) at 224 mOsm/kg]. Thus, the MX3 performed well at mid-ranges of osmolality but should be used with caution at extreme ranges of osmolality as the device tended to both overestimate (lower limit) and underestimate (upper limit) osmolality when compared to our criterion measure. When salivary osmolality thresholds are used for dehydration screening, the observed underestimation at higher osmolality values may have important implications. For instance, if an elevated salivary osmolality value is used to identify individuals at greater risk of dehydration, the M3′s underestimation of osmolality could result in classification below a screening threshold despite higher osmolality values measured by a laboratory osmometer, leading to a false-negative classification at the upper end of the measurement range. Therefore, when assessing individuals who are potentially severely dehydrated and for whom precise quantification is required, MX3 absolute values should be interpreted cautiously, and measurement with a laboratory-based osmometer may be warranted.

The proportional bias identified in this study aligns with previous studies measuring saliva osmolality and other field-based hydration measures (5, 8). For instance, Walsh et al. (5) demonstrated that disagreement between saliva and plasma osmolality increased when dehydration was equal to 2% body mass loss or greater. Taylor et al. (8) found that saliva osmolality and urine hydration markers increased proportional error compared to plasma osmolality during more severe dehydration states. Saliva and plasma osmolality in these studies were measured with laboratory-based freezing-point depression osmometry. Furthermore, Winter et al. found proportional bias when assessing the reliability of the MX3 device itself, especially at higher osmolality values (9).

Saliva has been recognized as a clinically useful point-of-care diagnostic fluid, as it can be collected non-invasively and is sensitive to changes in body water status (20). However, methodological differences in quantifying saliva osmolality between devices may explain the observed proportional bias in this study. The benchtop osmometer measures osmolality using freezing-point depression, while the MX3 system measures osmolality with electrochemical sensing. These methods differ since freezing point osmometry measures the total colligative solute concentration (i.e., total dissolved solutes regardless of chemical nature as ions or proteins) (6) while the electrochemical sensor measure responds to ionic conductivity and saliva composition, including the electrolyte distribution and protein concentration (5, 13). When dehydration severity increases, multiple changes occur that may alter the saliva's electrochemical properties independent of total osmolality; those changes include decreased saliva flow rate, increased total protein concentration, increased viscosity, and less uniform concentrations of sodium, chloride, and bicarbonate (3). Thus, the dehydration-induced changes in saliva composition may play a part in the proportional bias between devices, particularly at higher osmolarities.

The findings from this study suggest that the MX3 system may provide a practical field-based method for measuring salivary osmolality. However, this study evaluated agreement with a laboratory osmometer and within-sample reliability rather than the device's ability to monitor changes over time. The MX3 system demonstrated excellent within-sample reliability and moderate agreement with a laboratory-grade osmometer for measuring salivary osmolality. Proportional bias was observed at the extremes of the observed osmolality range, with the MX3 tending to overestimate lower osmolality values and underestimate higher ones relative to the benchtop osmometer. Therefore, absolute MX3 values should be interpreted carefully at the lower and upper ends of the measurement range. Hydration classification and diagnostic accuracy were not evaluated in this study, so using it to determine precise hydration status should be done with caution.

### Strengths and limitations

A primary strength of this study involved assessing the validity of the MX3 hydration system against the criterion freezing-point depression method across a variety of hydration scenarios commonly experienced in field and first-responder settings (i.e., during and following physical activity, and during sedentary work). Thus, our methodology demonstrates strong external validity.

Despite our strong methodological approach, several limitations exist for the present study. First, the devices required different saliva volumes to quantify osmolality. The benchtop osmometer required a significantly larger volume than the MX3 system, which may increase the differences in saliva composition being measured by each device, particularly at more pronounced dehydration states. Second, the saliva samples were pipetted onto the MX3 test strips, whereas the instructions for the device encourage the test strip to be tapped against the tongue for saliva collection. A tongue-tap collection method would be the typical field use method for the MX3 device, specifically. The composition of saliva may be different between the tongue-tap method and our method of collecting saliva in a test tube and pipetting it onto a strip, thus potentially adding to measurement variability. Notably, both approaches for MX3 saliva processing were tested within the laboratory (unpublished data) with no difference in osmolality values between pipetting or tongue tapping. Fourth, there was no standardized approach to collecting saliva samples from participants (e.g., passive drool method) (3). Finally, 80 paired samples were analyzed; however, these samples were obtained from 10 participants and therefore do not represent 80 independent samples. The absence of an a priori sample size calculation and the relatively small participant sample should be considered when interpreting the precision and generalizability of the agreement estimates. Furthermore, all participants were male recruit firefighters and represented a relatively healthy, physically active population that may be exposed to greater physical exertion, heat stress, and fluid loss than the general population. While our study population was not representative of a more diverse population, we believe these results are still generalizable as this study examines a device's capability of assessing hydration status; the MX3 measurement capabilities are not dependent upon population as it is intended to measure the solute of a fluid sample provided.

## Conclusions

Overall, our study demonstrated that the MX3 system had moderate agreement and strong consistency compared to a laboratory-grade freezing-point depression osmometer. Agreement between devices was strongest in the mid-range of osmolality values, while proportional bias was observed at the lower and higher values. These findings support using the MX3 as a practical, field-based method for measuring salivary osmolality when laboratory-based osmometry is unavailable. At higher saliva osmolality values, absolute measurements obtained from the MX3 should be used with caution, as they tend to underestimate osmolality relative to the laboratory osmometer. Additional research is needed to determine the device's efficacy in hydration screening, hydration status classification, and field-based decision-making.

## Data Availability

The raw data supporting the conclusions of this article will be made available by the authors, without undue reservation.

## References

[1] Armstrong LE. Assessing hydration status: the elusive gold standard. J Am Coll Nutr. (2007) 26:575S−84S. doi: 10.1080/07315724.2007.10719661 17921468

[2] Lindsey B Shaul Y Martin J. Salivary biomarkers of tactical athlete readiness: a systematic review. PLoS ONE. (2025) 20:e0321223. doi: 10.1371/journal.pone.0321223 40299918 PMC12040155

[3] Walsh NP Montague JC Callow N Rowlands AV. Saliva flow rate, total protein concentration and osmolality as potential markers of whole body hydration status during progressive acute dehydration in humans. Arch Oral Biol. (2004) 49:149–54. doi: 10.1016/j.archoralbio.2003.08.001 14693209

[4] Francisco R Jesus F Di Vincenzo O Nunes CL Alvim M Sardinha LB . Assessment of exercise-induced dehydration in underhydrated athletes: which method shows the most promise? Clin Nutr. (2024) 43:2139–48. doi: 10.1016/j.clnu.2024.08.003 39137516

[5] Walsh NP Laing SJ Oliver SJ Montague JC Walters R Bilzon JLJ. Saliva parameters as potential indices of hydration status during acute dehydration. Med Sci Sports Exerc. (2004) 36:1535–42. doi: 10.1249/01.mss.0000139797.26760.06 15354035

[6] Muñoz CX Johnson EC DeMartini JK Huggins RA McKenzie AL Casa DJ . Assessment of hydration biomarkers including salivary osmolality during passive and active dehydration. Eur J Clin Nutr. (2013) 67:1257–63. doi: 10.1038/ejcn.2013.195 24129362

[7] Zhao X McKenna ZJ Perez RI Wierick SC Allen BM McDermott BP. Assessment of the MX3 hydration testing system in adults at rest and following exercise in the heat. Physiol Rep. (2026) 14:e70757. doi: 10.14814/phy2.70757 41885769 PMC13097422

[8] Taylor NAS van den Heuvel AMJ Kerry P McGhee S Peoples GE Brown MA . Observations on saliva osmolality during progressive dehydration and partial rehydration. Eur J Appl Physiol. (2012) 112:3227–37. doi: 10.1007/s00421-011-2299-z 22230919

[9] Winter I Burdin J Wilson PB. Reliability and minimal detectable change of the MX3 hydration testing system. PLoS ONE. (2024) 19:e0313320. doi: 10.1371/journal.pone.0313320 39570831 PMC11581297

[10] Kwan P. Independent Validation of Salivary Osmolarity Measures made by the MX3 Hydration Testing System Compared to a Laboratory Grade Osmometer (2019). Available online at: https://mx3diagnostics.com/files/files/au/z2_independent-validation-report.pdf (Accessed February 10, 2026).

[11] Faidah N Soraya GV Erlichster M Natzir R Chana G Skafidas E . Detection of voluntary dehydration in paediatric populations using non-invasive point-of-care saliva and urine testing. J Paediatr Child Health. (2021) 57:813–8. doi: 10.1111/jpc.15325 33373495

[12] Atjo NM Soraya GV Natzir R Kasyim H Rasyid H Chana G . Point-of-care saliva osmolarity testing for the screening of hydration in older adults with hypertension. J Am Med Directors Assoc. (2022) 23:1984.e9–e14. doi: 10.1016/j.jamda.2022.08.015 36174654

[13] Mani V Beduk T Khushaim W Ceylan AE Timur S. Electrochemical sensors targeting salivary biomarkers: a comprehensive review. TrAC Trends Anal Chem. (2020) 116164. doi: 10.1016/j.trac.2020.116164

[14] Lawrence I-Kuei Lin. A concordance Correlation Coefficient to Evaluate Reproducibility. Biometrics. (1989) 45:255–68. doi: 10.2307/2532051 2720055

[15] Lakens D Scheel AM Isager PM. Equivalence Testing for Psychological Research: a Tutorial. Adv Methods chological Science. (2018) 1:259–69. doi: 10.1177/2515245918770963

[16] SHAPIRO SS WILK MB. An analysis of variance test for normality (complete samples). Biometrika. (1965) 52:591–611. doi: 10.1093/biomet/52.3-4.591

[17] Breusch TS Pagan AR. A simple test for heteroscedasticity and random coefficient variation. Econometrica. (1979) 47:1287–94. doi: 10.2307/1911963

[18] Donner A Zou G. Closed-form confidence intervals for functions of the normal mean and standard deviation. Stat Methods Med Res. (2012) 21:347–59. doi: 10.1177/0962280210383082 20826501

[19] Caldwell AR. SimplyAgree: An R package and jamovi module for simplifying agreement and reliability analyses. J Open Source Softw. (2022) 7:4148. doi: 10.21105/joss.04148

[20] Kalra S Sharma S Verma SK Thakor P Malve H Chamle V . Assessment of hydration status using conventional method and salivary osmolarity as a point-of-care tool. J Assoc Physicians India. (2024) 72:30–8. doi: 10.59556/japi.72.0545 38932733

